# A Rare Case of Extralobar Bronchopulmonary Sequestration

**DOI:** 10.7759/cureus.73330

**Published:** 2024-11-09

**Authors:** Nadezhda Tolekova, Zdravka Antonova, Nikola Kartulev, Natalia Gabrovska, Hristo Shivachev

**Affiliations:** 1 Pediatric Surgery, University Multi-Profile Hospital for Active Treatment and Emergency Medicine (UMHATEM) "N.I. Pirogov", Sofia, BGR; 2 Pediatric Pulmology, Specialized Hospital for Active Treatment of Children’s Diseases “Prof. Ivan Mitev”, Sofia, BGR

**Keywords:** bronchopulmonary sequestration, children, extralobar, intradiaphragmatic, surgery

## Abstract

Bronchopulmonary sequestration is a rare developmental abnormality of the pulmonary system. It is a pulmonary malformation and is defined as lung tissue without connection to the tracheobronchial tree with a systemic blood supply.

We present a case of an eight-month-old infant with prenatally diagnosed bronchopulmonary sequestration. At six months of age, an extralobar sequestration with a feeding vessel from truncus coeliacus and venous drainage to vena thoracica interna sinistra was diagnosed from a CT scan of the lung. Thoracoscopy was performed with the identification of intradiaphragmatic sequestration, conversion to thoracotomy with resection of the lesion, and repair of the diaphragm. Postoperative follow-up revealed no complications.

Intradiaphragmatic bronchopulmonary sequestration is an extremely rare congenital anomaly. The gold standard for diagnostic clarification is computed tomography. The preferred approach for surgical treatment is transthoracic.

## Introduction

Bronchopulmonary sequestration is a rare developmental abnormality of the pulmonary system. It accounts for 0.15-6.4% of all pulmonary malformations and is defined as lung tissue without connection to the tracheobronchial tree with a systemic blood supply [1]. Intradiaphragmatic pulmonary sequestration is extremely rare, accounting for 13-18% of all sequestrations. It was first described by Caulet in 1962 [2,3].

## Case presentation

**Figure 1:**
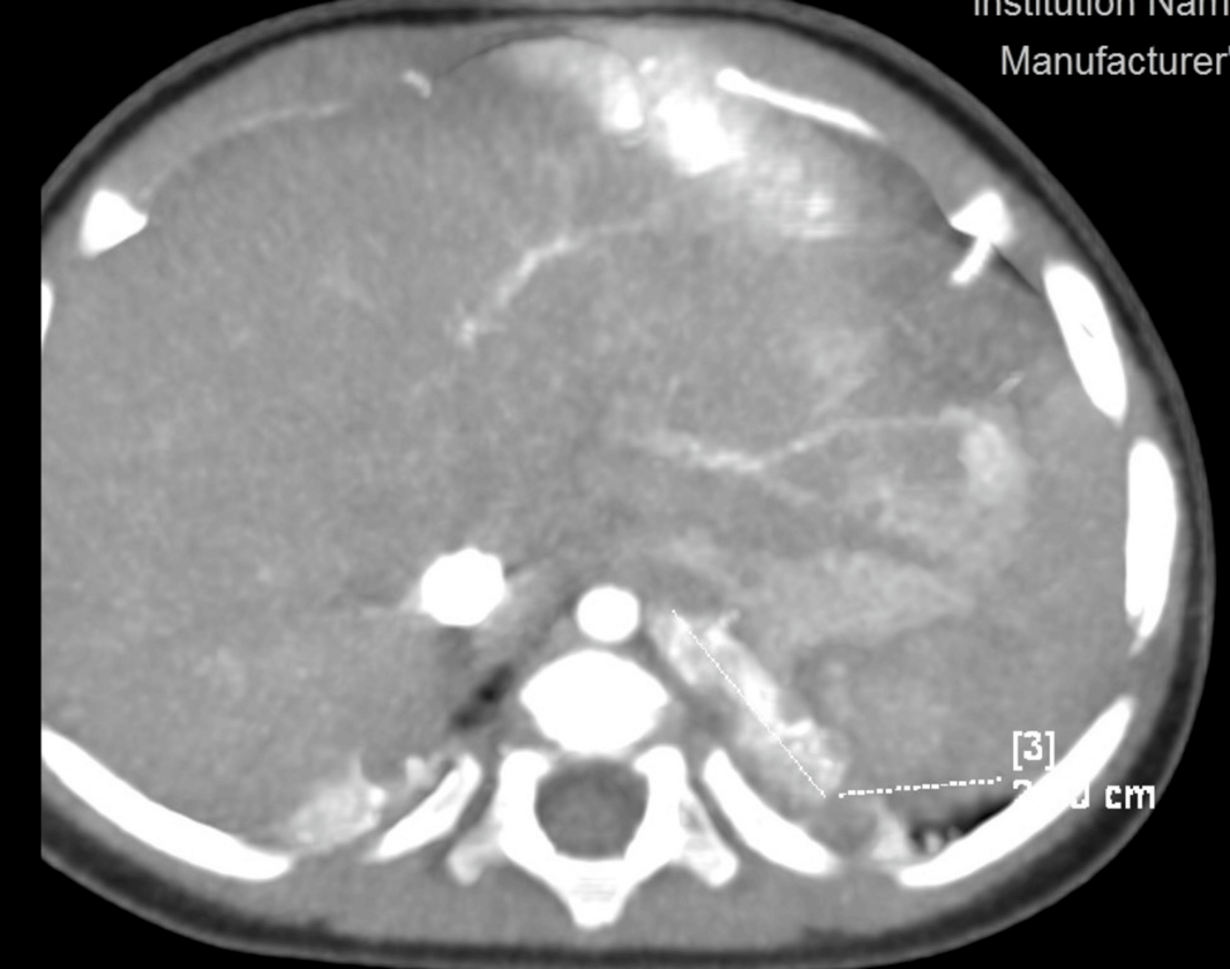
Computed tomography; position of the anomaly, axial

**Figure 2:**
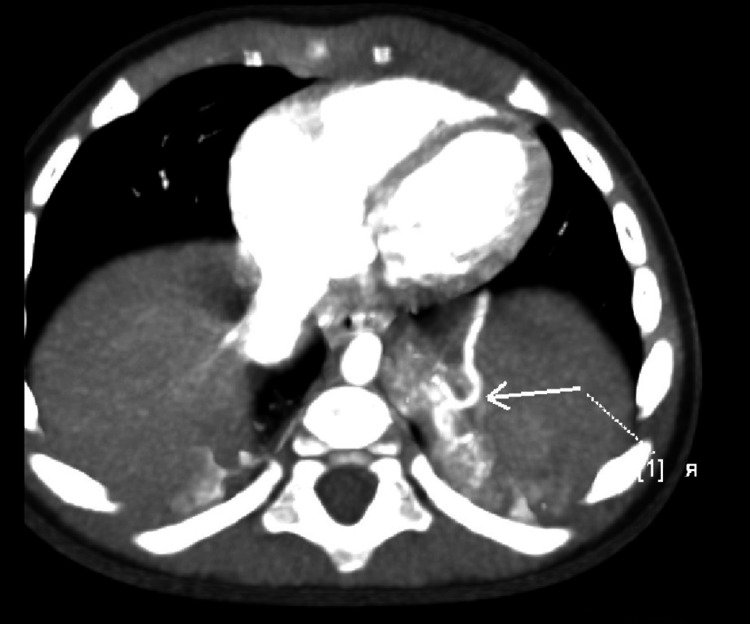
Computed tomography; feeding vessel from truncus coeliacus

We present a case of an eight-month-old infant from the first normal pregnancy of a mother with Hashimoto's thyroiditis. A left bronchopulmonary sequestration was suspected prenatally. The child was born five days post-term weighing 2950 g. Following birth, there were no signs of respiratory distress. No concomitant abnormalities were found. A chest CT was performed at six months of age, which revealed an extralobar sequestration on the left measuring 26/26/9 mm, located supradiaphragmatically with an arterial vessel arising from the truncus coeliacus and venous drainage to the vena thoracica interna sinistra (Figure 1, Figure 2).

**Figure 3:**
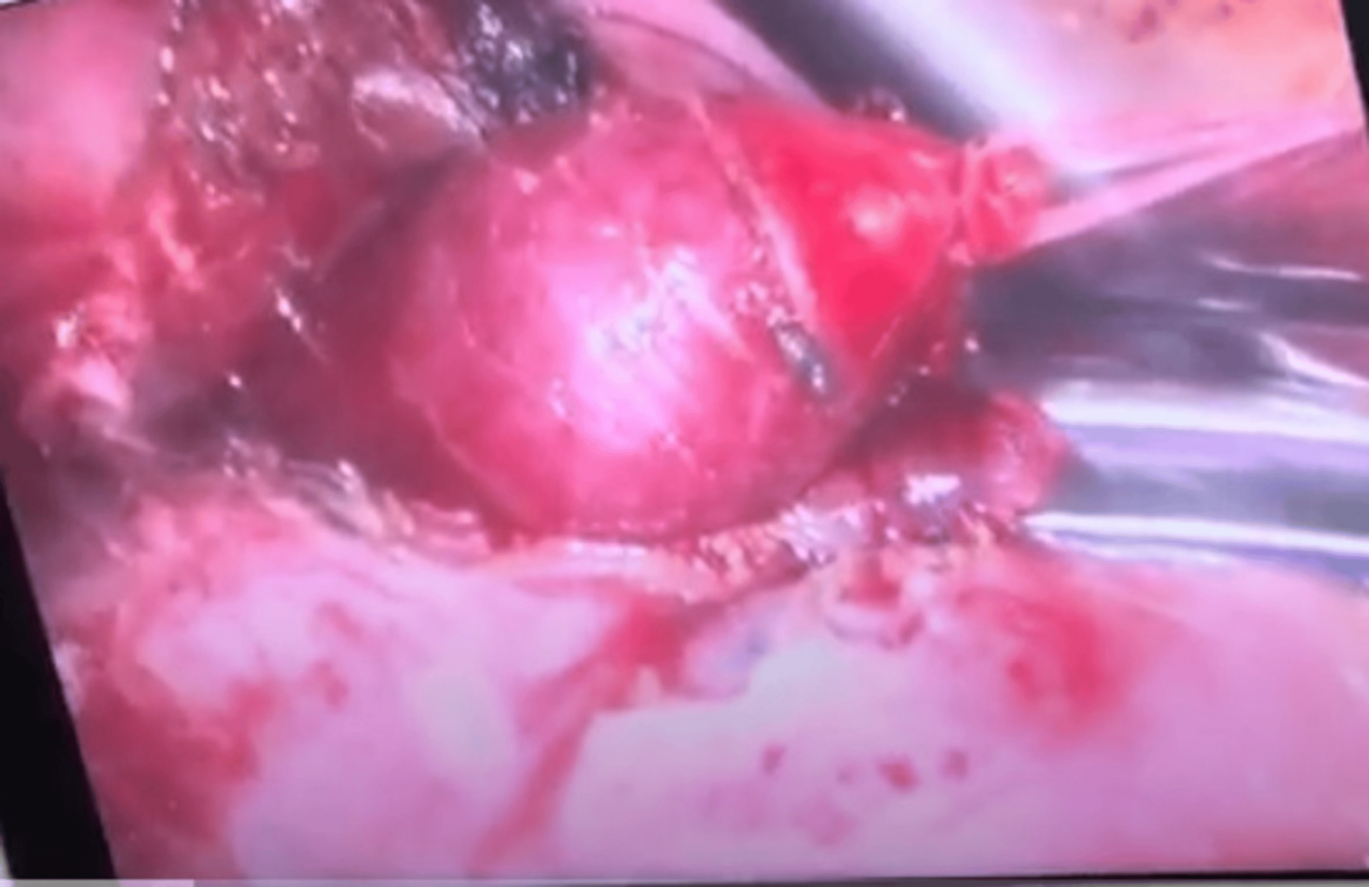
Intradiaphragmatic bronchopulmonary sequestration

**Figure 4:**
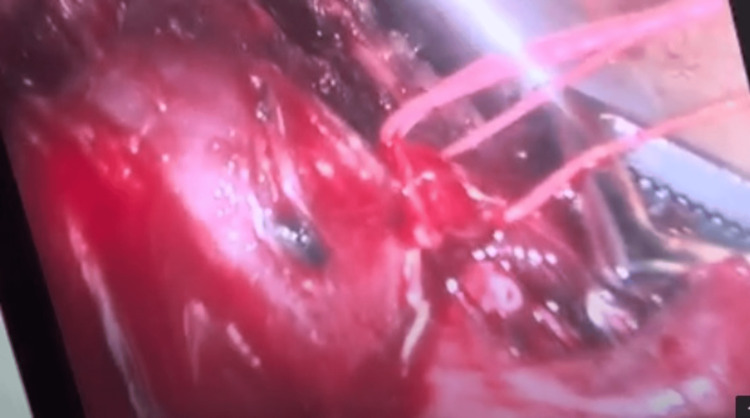
Ligation of the feeding vessel of the sequestration

At the age of eight months, surgical treatment was performed, a video-assisted thoracoscopy. Intraoperatively, the intradiaphragmatic location of the sequestration was found to be to the left posterolateral. Due to the inability to differentiate clear borders of the formation, conversion to posterior thoracotomy in the eighth intercostal space was performed. The sequestration was covered by a thin layer of diaphragmatic muscle fibers, which was opened. The feeding vessels were identified and ligated (Figure 3, Figure 4).

**Figure 5:**
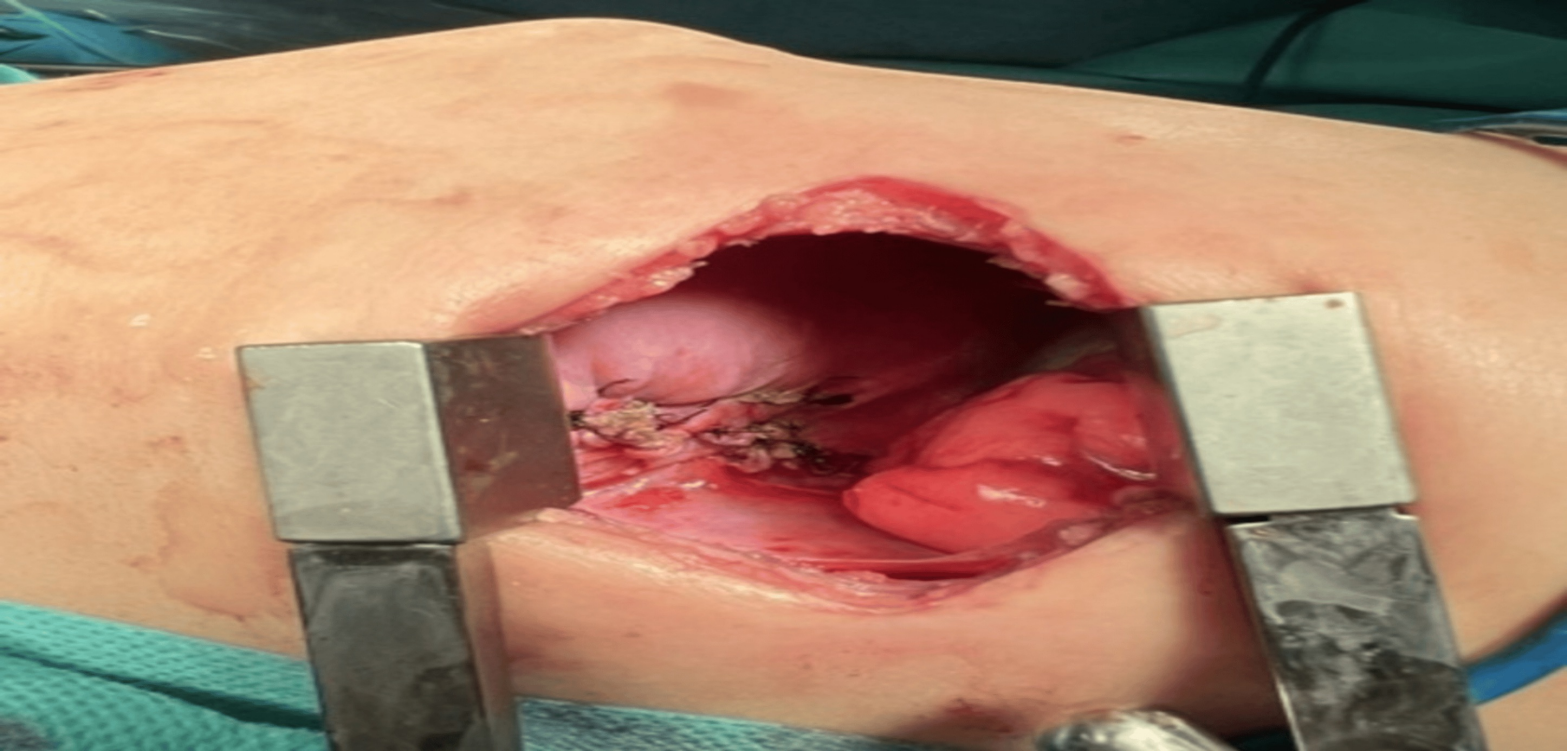
Plasty of the diaphragm after resection of the sequestration

The formation was dissected away from the diaphragmatic musculature, and subsequently, the integrity of the diaphragm was restored with single non-absorbable multifilament braided sutures in two layers (Figure 5).

**Figure 6:**
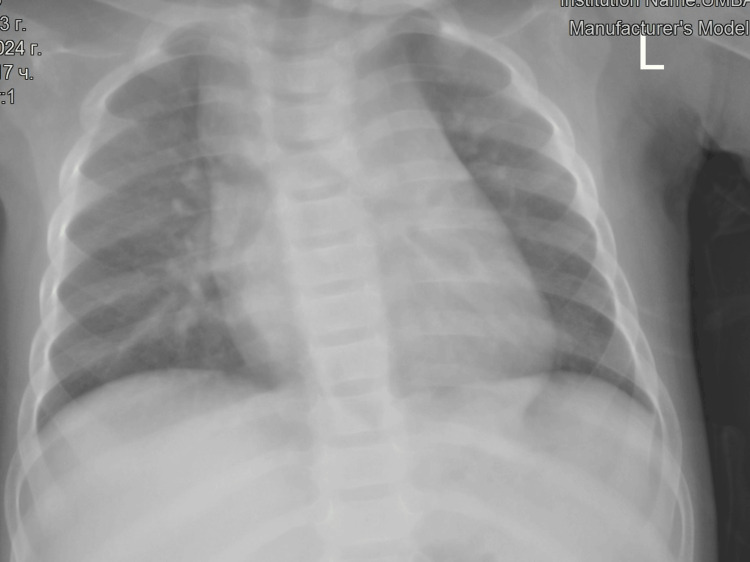
Pulmonary X-ray, one month after surgery

Postoperatively, increased inflammatory activity was observed, necessitating a change in antibiotic therapy, adding amikacin to the started ampicillin/sulbactam. The thoracic drain was removed on the second postoperative day. The child was discharged on postoperative day 9. The histological result confirmed bronchopulmonary sequestration. At nine-month follow-up, no complications were found, with no respiratory infections and normal diaphragmatic mobility (Figure 6).

## Discussion

Bronchopulmonary sequestration was first described by Rokitansky in 1861 [4]. There are multiple hypotheses for the etiology of bronchopulmonary sequestrations, the most common being the formation of an extrapulmonary bud between the 4th and 8th gestational weeks and a disturbance in the ratio of proliferation to apoptosis [4,5]. The early stage of onset determines the possibility of detecting combined anomalies: diaphragmatic hernia, cardiac malformation, bronchogenic cysts, and chest deformities [6,7]. According to the presence or absence of its own visceral pleura, bronchopulmonary sequestrations are classified as intralobar and extralobar. Intralobar sequestration is seen in about 75% of cases and is covered by the visceral pleura of the normal lung parenchyma and is localized in the lower lung lobes, more commonly on the left. Venous drainage of intralobar sequestration is most commonly via the pulmonary veins. Extralobar sequestrations (25%) are covered by their own pleura and can be located intrathoracically, intradiaphragmatic, and subdiaphragmatic, more commonly on the left. Venous drainage is usually via the vena azygos or vena hemiazygos, rarely via the inferior vena cava, vena subclavia, or portal vein. Usually, the arterial blood supply is anomalous from the descending portion of the thoracic aorta. In about 20% of cases, a systemic subdiaphragmatic arterial vessel is found in the extralobar sequestrations: abdominal aorta, truncus coeliacus, and splenic artery [8]. Extralobar intradiaphragmatic sequestration is extremely rare. It is thought to develop as a result of the fusion of the accessory pulmonary bud with the embryonic elements of the diaphragm [9]. With the development of prenatal diagnosis, the incidence of sequestrations detected by prenatal ultrasonography at the earliest at the 16th week of gestation has increased, presenting as a solid, well-circumscribed, homogeneous, hyperechogenic formation [10,11]. In a small percentage of cases, the formation is detected incidentally on examination on another occasion [12]. However, the specific intradiaphragmatic localization is rarely confirmed by ultrasonography. In a report by Gao et al., only 40% of cases of prenatal ultrasonography identified the exact location [13]. For a more detailed evaluation of the abnormality, magnetic resonance imaging (MRI) and/or computed tomography (CT) are performed. MRI does not use ionizing radiation and provides detailed information in evaluating the vessels of the malformation, but the diaphragm may be affected by respiratory movements during the study, limiting its use for diagnosing intradiaphragmatic location of sequestration [14]. CT is less affected by the respiratory motion of the diaphragm and is the most accurate method for detecting systemic vascularization of the formation [13]. Gao et al. presented 10 cases of intradiaphragmatic localization from 215 operated patients with bronchopulmonary sequestration over a five-year period [13]. Their reported concordance rate between preoperative CT and intraoperative findings was 70%. For the first time, they also presented a 3D reconstruction to further clarify the anatomy and plan the surgical treatment [13]. In the present case, the diagnosis was made prenatally and confirmed at six months of age by performing a CT scan.

Postnatally, bronchopulmonary sequestrations may be asymptomatic or manifest with dyspnea, cyanosis, chronic cough, wheezing, and recurrent pneumonia [15]. In the long term, inflammatory complications or malignant degeneration may develop. There is no uniform opinion regarding therapeutic management. Some studies suggest that extralobar sequestrations may remain asymptomatic throughout life and even involute, suggesting the safety of dynamic follow-up [16,17]. However, most surgeons advise that sequestrations should be resected by one year of age [1,6,17]. A study by Trabalza Marinucci et al. indicated that patients operated on after one year of age developed more respiratory complications [1], and Stanton found a higher rate of postoperative complications in symptomatic patients [18]. Our clinic maintains the recommendation for operative treatment of bronchopulmonary sequestrations before the age of one year. In the case described, surgical treatment was performed at eight months of age.

Intradiaphragmatic bronchopulmonary sequestrations are challenging both for diagnosis and for the choice of surgical approach. Some authors favor the thoracoscopic approach [2,13], while others find identification of the lesion easier by laparoscopy [19]. There are also reports of combined approaches [3,15]. The use of transcatheter arterial embolization has also been reported, but this method is not widespread and is accompanied by many complications [20]. Gao et al. reported for the first time the use of robotic thoracoscopy for intradiaphragmatic sequestration resection in four patients, which they said provided image enhancement, gave more flexibility and precision of performance, but was associated with higher costs and a complete lack of tactile sensation [13]. In the reported case, surgical intervention was initiated thoracoscopically, but the lack of a clear margin led to the need for conversion.

## Conclusions

Intradiaphragmatic bronchopulmonary sequestration is a very rare congenital condition. The advent of prenatal ultrasonography has enabled its early detection. Further detailed information about the anatomical relationships and vascular supply can be obtained through contrast-enhanced computed tomography following birth. The risk of inflammatory complications and potential malignant transformation underscores the importance of early surgical intervention. The optimal surgical approach is thoracic, which is associated with low morbidity and mortality rates.

## References

[1] Trabalza Marinucci B, Maurizi G, Vanni C (2020). Surgical treatment of pulmonary sequestration in adults and children: long-term results. Interact Cardiovasc Thorac Surg.

[2] Chun H, Gang Y, Xiaochun Z, Jin T, Bo X, Limin W, Cuifen L (2015). Diagnosis and management of intradiaphragmatic extralobar pulmonary sequestration: a report of 11 cases. J Pediatr Surg.

[3] Nijagal A, Jelin E, Feldstein VA (2012). The diagnosis and management of intradiaphragmatic extralobar pulmonary sequestrations: a report of 4 cases. J Pediatr Surg.

[4] Abuhamad AZ, Bass T, Katz ME, Heyl PS (1996). Familial recurrence of pulmonary sequestration. Obstet Gynecol.

[5] Sade R, Clouse M, Ellis F (1974). The spectrum of pulmonary sequestration. Ann Thorac Surg.

[6] Dell'Amore A, Giunta D, Campisi A (2018). Uniportal thoracoscopic resection of intralobar and extralobar pulmonary sequestration. J Vis Surg.

[7] Corbett HJ, Humphrey GM (2004). Pulmonary sequestration. Paediatr Respir Rev.

[8] Lager DJ, Kuper KA, Haake GK (1991). Subdiaphragmatic extralobar pulmonary sequestration. Arch Pathol Lab Med.

[9] Oreglio C, Tocchioni F, Ghionzoli M, Buccoliero A, Morabito A, Morini F (2023). Intradiaphragmatic pulmonary sequestrations: a surgical challenge. Case series. Front Surg.

[10] Bentur L, Gur M, Pollak M, Masarweh K, Solt I, Bronshtein M (2019). Early prenatal ultrasound diagnosis of congenital thoracic malformations. J Matern Fetal Neonatal Med.

[11] Costa MR, Costa TR, Leite MS, Filho FR, Reis AM, Pereira BP, Oliveira AM (2016). Atypical presentation of intra-abdominal extralobar pulmonary sequestration detected in prenatal care: a case report (Article in Portuguese). Rev Paul Pediatr.

[12] Gudavalli R, Farver CC, Mason DP, Hatipoglu U (2015). Intralobar pulmonary sequestration presenting as chronic non-productive cough. Indian J Chest Dis Allied Sci.

[13] Gao Y, Han X, Jin J, Tan Z (2022). Ten cases of intradiaphragmatic extralobar pulmonary sequestration: a single-center experience. World J Pediatr Surg.

[14] Mon RA, Johnson KN, Ladino-Torres M, Heider A, Mychaliska GB, Treadwell MC, Kunisaki SM (2019). Diagnostic accuracy of imaging studies in congenital lung malformations. Arch Dis Child Fetal Neonatal Ed.

[15] Ramos AK, Teramae TR, Liang CG, Miriam L. Brazer ML, Woo RK (2023). The complexities in diagnosing intradiaphragmatic extrapulmonary sequestration: a case report. Ann Pediatr Surg.

[16] Laberge JM, Puligandla P, Flageole H (2005). Asymptomatic congenital lung malformations. Semin Pediatr Surg.

[17] Robson VK, Shieh HF, Wilson JM, Buchmiller TL (2020). Non-operative management of extralobar pulmonary sequestration: a safe alternative to resection?. Pediatr Surg Int.

[18] Stanton M (2015). The argument for a non-operative approach to asymptomatic lung lesions. Semin Pediatr Surg.

[19] Schuster B, Holland-Cunz SG, Zimmermann P (2017). Intradiaphragmatic hybrid lesion: surgical decision-making and value of minimal invasive surgery. BMJ Case Rep.

[20] Cho MJ, Kim DY, Kim SC, Kim KS, Kim EA, Lee BS (2012). Embolization versus surgical resection of pulmonary sequestration: clinical experiences with a thoracoscopic approach. J Pediatr Surg.

